# Impact of COVID-19 pandemic on catheter ablation in China: A spatiotemporal analysis

**DOI:** 10.3389/fpubh.2022.1027926

**Published:** 2022-11-23

**Authors:** Jiang Jiang, Shuang Zhao, Chendi Cheng, Na Lin, Ping Li, Xiaohui Ning, Shu Zhang

**Affiliations:** ^1^Arrhythmia Center, State Key Laboratory of Cardiovascular Disease, Fuwai Hospital, National Center for Cardiovascular Diseases, Chinese Academy of Medical Sciences and Peking Union Medical College, Beijing, China; ^2^National Center for Cardiovascular Quality Improvement Committee, Fuwai Hospital, Beijing, China; ^3^Chinese Society of Arrhythmia, Beijing, China; ^4^Chinese Society of Pacing and Electrophysiology, Beijing, China

**Keywords:** COVID-19, pandemic, China, catheter ablation, Arrhythmia

## Abstract

**Background:**

The COVID-19 pandemic has significantly impacted routine cardiovascular health assessments and services. We aim to depict the temporal trend of catheter ablation (CA) and provide experience in dealing with the negative impact of the COVID-19 pandemic.

**Methods:**

Data on CA between January 2019, and December 2021, were extracted from the National Center for Cardiovascular Quality Improvement platform. CA alterations from 2019 to 2021 were assessed with a generalized estimation equation.

**Results:**

A total of 347,924 patients undergoing CA were included in the final analysis. The CA decreased remarkably from 122,839 in 2019 to 100,019 (−18.58%, 95% CI: −33.40% to −3.75%, *p* = 0.02) in 2020, and increased slightly to 125,006 (1.81%, 95% CI: −7.01% to 3.38%, *p* = 0.49) in 2021. The CA experienced the maximal reduction in February 2020 (−88.78%) corresponding with the peak of monthly new COVID-19 cases and decreased by 54.32% (95%CI: −71.27% to −37.37%, *p* < 0.001) during the 3-month lockdown and increased firstly in June 2020 relative to 2019. Since then, the CA in 2020 remained unchanged relative to 2019 (−0.06%, 95% CI: −7.01% to 3.38%, *p* = 0.98). Notably, the recovery of CA in 2021 to pre-COVID-19 levels was mainly driven by the growth of CA in secondary hospitals. Although there is a slight increase (2167) in CA in 2021 relative to 2019, both the absolute number and proportion of CA in the top 50 hospitals nationwide [53,887 (43.09%) vs. 63,811 (51.95%), *p* < 0.001] and top three hospitals in each province [66,152 (52.73%) vs. 72,392 (59.28%), *p* < 0.001] still declined significantly.

**Conclusions:**

The CA experienced a substantial decline during the early phase of the COVID-19 pandemic, and then gradually returned to pre-COVID-19 levels. Notably, the growth of CA in secondary hospitals plays an important role in the overall resumption, which implies that systematic guidance of secondary hospitals with CA experience may aid in mitigating the negative impact of the COVID-19 pandemic.

## Introduction

The Coronavirus Disease 2019 (COVID-19) pandemic has posed an unprecedented health threat and varying degrees of negative effects on healthcare systems worldwide (1, 2). Limited medical resources were reorganized to prevent the spread of the COVID-19 pandemic and ensure the availability of emergency medical care. Despite this, there was a substantial decline in medical care for several urgent cases, such as delays in the emergency intervention for acute coronary syndrome (3–6). As a consequence, non-emergency procedures had been hit harder (7–9).

Several studies have reported varying degrees of reduction in catheter ablation (CA) volume ranging from 21 to 83% in the early phase of the COVID-19 outbreak (7, 10). In the context of the long-term and ongoing COVID-19 pandemic, it is of great significance to understand the subsequent changes in CA volume and possible mitigation measures. In theory, the improved understanding of severe acute respiratory syndrome coronavirus 2 (SARS-CoV-2), extensive use of nucleic acid detection, COVID-19 drugs and vaccines, effective personal protective equipment, and others may have alleviated the negative shocks of the pandemic (11–14). Moreover, several guidance has been published by the European Society of Cardiology (15) and the American College of Cardiology (16) in response to the negative impact of the COVID-19 pandemic on electrophysiological procedures. In addition, the Chinese Society of Arrhythmia (CSA) and the Chinese society of pacing and electrophysiology (CSPE) jointly launched the “3R Telemedicine Project” in the second half of 2020. The project aimed to improve the ability of electrophysiologists in secondary hospitals via remote training, real-time procedures guidance, and follow-up. However, the extent to which healthcare systems have adapted to the COVID-19 pandemic to provide routine medical services for CA is unknown. Therefore, this study was designed to depict the temporal trend of CA and provide experience in dealing with the negative impact of the COVID-19 pandemic on CA with the data from the National Center for Cardiovascular Quality Improvement (NCCQI) platform.

## Methods

### Data sources and populations

We extracted data on CA from the NCCQI platform, established by the National Health Commission of the People's Republic of China, to improve and standardize cardiology clinical practice (17). Patients who underwent CA between January 1, 2019, to December 31, 2021, across 30 provinces (except Tibet, Hong Kong, Macau, and Taiwan) in China due to atrial fibrillation (AF), atrial flutter (AFL), atrial tachycardia (AT), paroxysmal supraventricular tachycardia (PSVT), ventricular tachycardia (VT), or premature ventricular contraction (PVC) were included in this analysis. The number of new and cumulative confirmed COVID-19 cases in the Chinese mainland were obtained from the China Center of Disease Control and used for non-commercial under the Creative Commons by Attribution Non-Commercial 4.0 International License (18). This study conformed to the Declaration of Helsinki and the need for approval was waived, given the nature of the data, after consulting the institutional ethics committee. All datasets were de-identified before data analysis to protect the patients' privacy.

To explore whether there were distributional disparities in CA volume at different levels of the hospitals in 2019, 2020, and 2021, hospitals registered on the NCCQI platform were divided into major and secondary centers. The major centers referred to the top three hospitals in each province based on the CA volume and the remaining hospitals were defined as the secondary centers. Qinghai and Hainan provinces were excluded from this analysis because they had no more than three hospitals registered on the NCCQI platform.

### Statistical analysis

Data distribution was explored for normality using the Shapiro–Wilk test and data with skewness distribution were described as median (M) with interquartile ranges (IQRs). Categorical variables were presented as frequency (percentage) and compared with the χ^2^ or Fisher exact tests, as appropriate. A generalized estimation equation was established to assess the difference in monthly and regional CA volume in 2019, 2020, and 2021 under the assumption that the number of CA follows a Poisson distribution with a log link function (19). The number of new COVID-19 cases was logarithm transformed to normalize the distribution. A scatter plot and simple linear regression model were used to investigate the association between CA volume alterations and the corresponding number of new COVID-19 cases. For all tests, a two-sided *p*-value < 0.05 was considered statistically significant. All data were analyzed with the GraphPad Prism (version 8.2.1, San Diego, CA) and R software Version 3.5.3 (R Foundation for Statistical Computing, Vienna, Austria).

## Results

**Figure 1:**
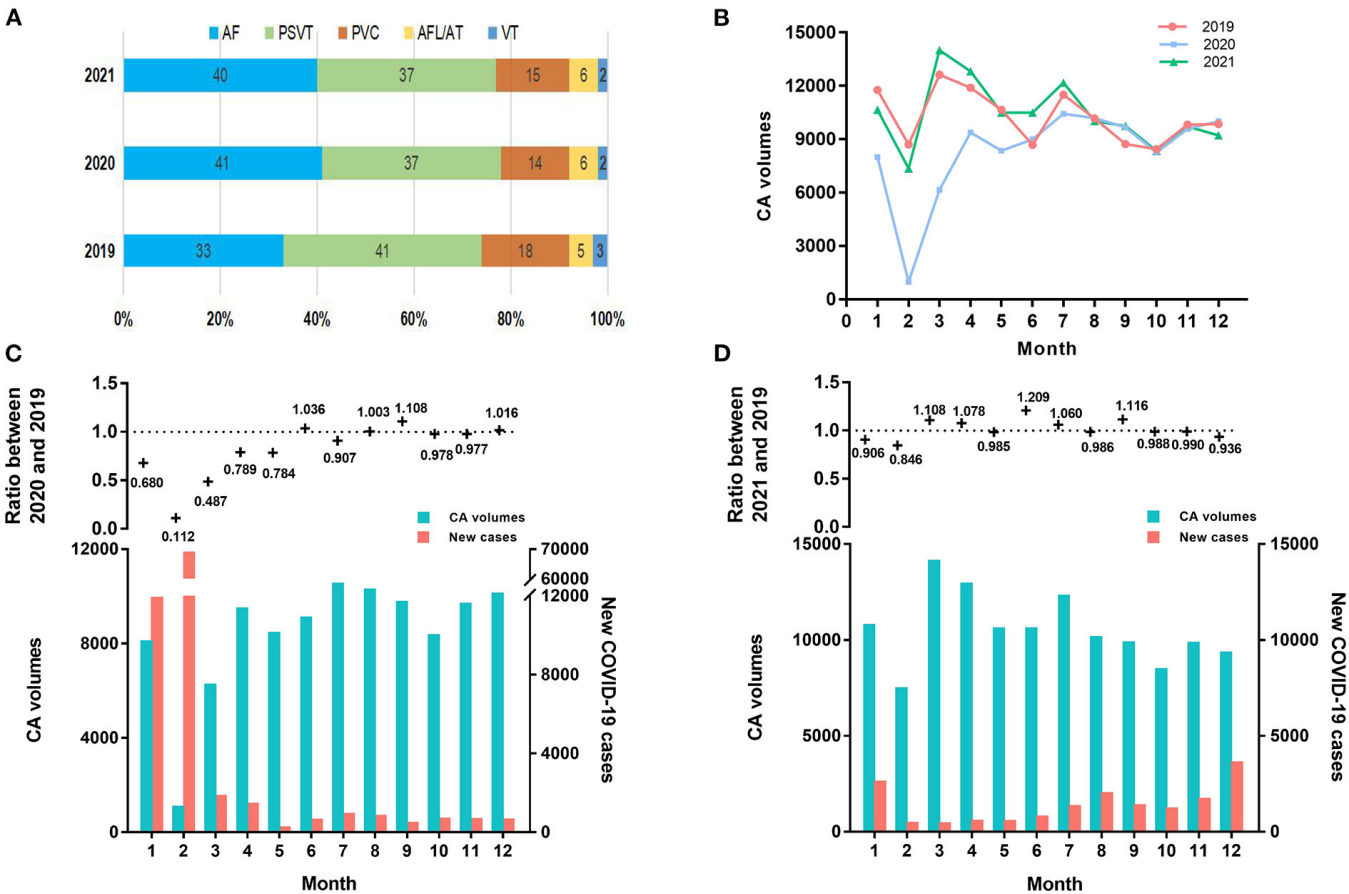
The dynamic alterations of CA volume and COVID-19 cases. **(A)** Etiological composition of CA in 2019, 2020, and 2021. **(B)** Monthly number of CA in 2019, 2020, and 2021. **(C)** In the upper panel, the ratio of the monthly number of CA in 2020 relative to 2019 was shown. In the lower panel, the monthly number of CA and new COVID-19 cases in 2020 were shown. **(D)** In the upper panel, the ratio of the monthly number of CA in 2021 relative to 2019 was shown. In the lower panel, the monthly number of CA and new COVID-19 cases in 2021 were shown. AF, atrial fibrillation; AFL, atrial flutter; AT, atrial tachycardia; CA, catheter ablation; COVID-19, Coronavirus Disease 2019; PSVT, paroxysmal supraventricular tachycardia; PVC, premature ventricular contraction; VT, ventricular tachycardia.

From January 1, 2019, to December 31, 2021, a total of 347,924 CA from 1,130 hospitals in 30 Chinese provinces were registered on the NCCQI platform. The CA volume decreased significantly from 122,839 in 2019 to 100,019 in 2020 and slightly increased to 125,006 in 2021; on average, PSVT (38.4%) was the most common, followed by AF (37.82%). Figure 1A shows more detailed information on this.

### Monthly CA volume alterations from 2019 to 2021

**Figure 2:**
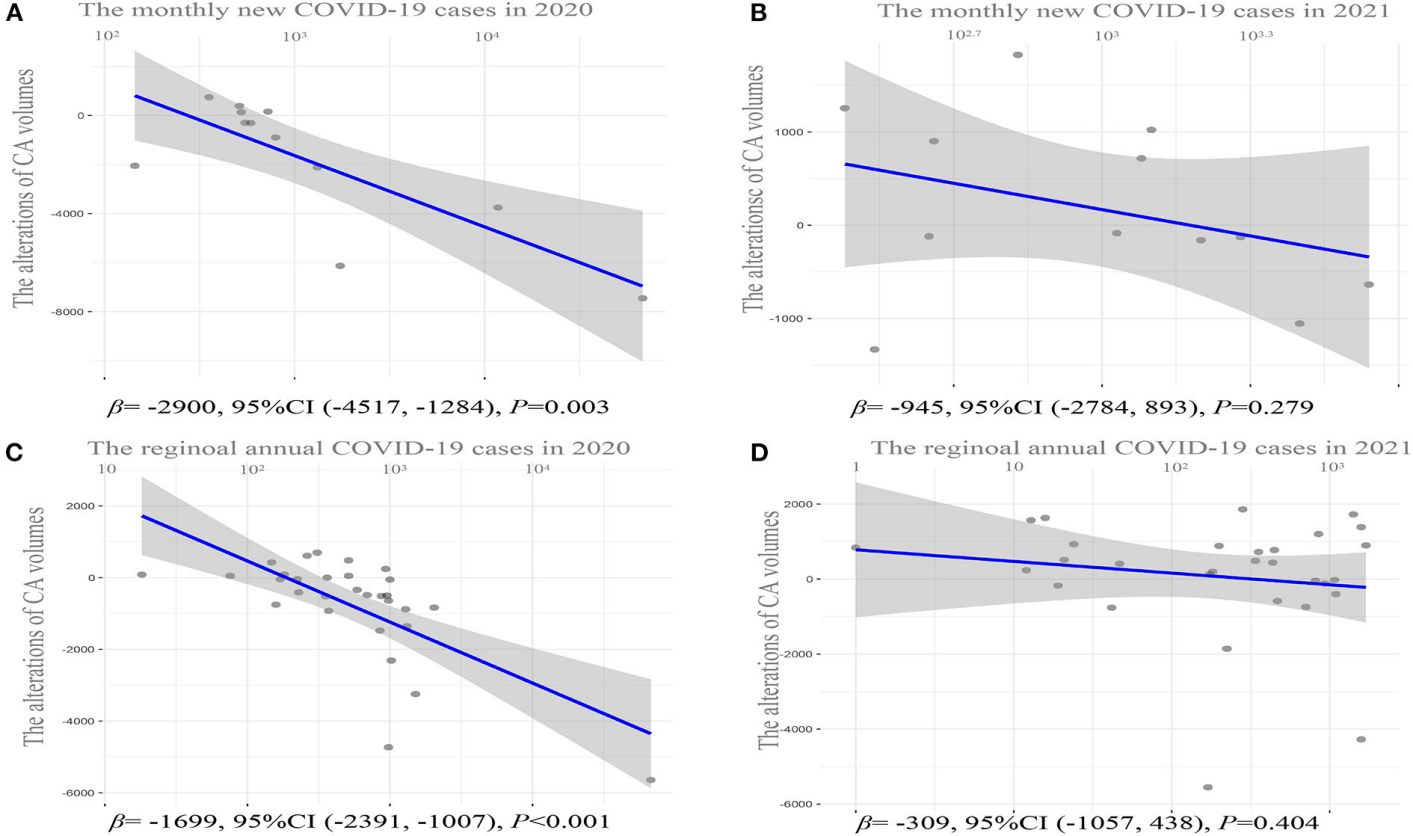
Correlation analysis of CA volume alterations in 2020 or 2021 relative to 2019 and the corresponding number of new COVID-19 cases. **(A)** Scattered plot for monthly CA volume alterations in 2020 relative to 2019 and the corresponding number of new COVID-19 cases in 2020. **(B)** Scattered plot for monthly CA volume alterations in 2021 relative to 2019 and the corresponding number of new COVID-19 cases in 2021. **(C)** Scattered plot for annual CA volume alterations in 2020 relative to 2019 and the corresponding number of new COVID-19 cases in 2020 in each province. **(D)** Scattered plot for annual CA volume alterations in 2021 relative to 2019 and the corresponding number of new COVID-19 cases in 2021 in each province. CA, catheter ablation; COVID-19, Coronavirus Disease 2019.

Table 1 shows the monthly number of CA or new COVID-19 cases in 2019, 2020, and 2021. The CA volume in 2020 declined significantly by 18.58% (95% confidence interval (CI): −33.40% to −3.75%, *p* = 0.02) relative to 2019. Particularly, the CA volume experienced the m reduction in February 2020 corresponding with the peak of the monthly number of new COVID-19 cases (Table 1). Meanwhile, the CA volume decreased by 54.32% (95% CI: −71.27% to −37.37%, *p* < 0.001) during January and March 2020, when a nationwide lockdown was implemented (Figures 1B,C). After keeping the average daily reported COVID-19 cases of 50 or less for 2 months, the CA volume increased firstly in June 2020 relative to the same period in 2019. Since then, the CA volume in 2020 remained unchanged compared to the same period in 2019 (−0.06%, 95% CI: −4.31% to 4.42%, *p* = 0.98). Meanwhile, 87,071 COVID-19 cases were reported in 2020. Furthermore, there was a significant inverse correlation (β = −2900, 95% CI: −4517 to −1284, *p* = 0.003) between the monthly CA volume alterations in 2020 relative to 2019 and the monthly number of new COVID-19 cases in 2020, which means that the monthly CA volume decreased by 2900 on average per 10-fold increase in the monthly number of COVID-19 cases (Figure 2A).

**Table 1:** Monthly CA volume or new COVID-19 cases in 2019, 2020, and 2021.

**Month**	**2019**	**2020**			**2021**		
		**CA volume**	**Changes %**	**New cases (%)**	**CA volume**	**Changes %**	**New cases (%)**
1	11,764	7,996	−32.03%	11,791 (13.54%)	10,655	−9.43%	2,493 (16.36%)
2	8,702	976	−88.78%	68,033 (78.14%)	7,361	−15.41%	348 (2.28%)
3	12,637	6,150	−51.34%	1,730 (1.99%)	14,006	10.83%	305 (2%)
4	11,890	9,386	−21.06%	1,320 (1.52%)	12,819	7.81%	454 (2.98%)
5	10,651	8,353	−21.58%	143 (0.16%)	10,492	−1.49%	451 (2.96%)
6	8,679	8,995	3.64%	517 (0.59%)	10,490	20.87%	670 (4.4%)
7	11,501	10,436	−9.26%	803 (0.92%)	12,187	5.96%	1,213 (7.96%)
8	10,169	10,197	0.28%	721 (0.83%)	10,023	−1.44%	1,893 (12.42%)
9	8,731	9,674	10.80%	356 (0.41%)	9,747	11.64%	1,264 (8.29%)
10	8447	8258	−2.24%	583 (0.67%)	8,348	−1.17%	1,081 (7.09%)
11	9,814	9,584	−2.34%	545 (0.63%)	9,716	−1.00%	1,581 (10.37%)
12	9,854	10,015	1.64%	529 (0.61%)	9,222	−6.41%	3,490 (22.9%)
Total	122,839	100,019	−18.58%^a^	87,071 (100%)	125,066	1.81%^b^	15,243 (100%)

^a^In comparison with the same period in 2019, the CA volume alterations in 2020 were statistically significant (*p* = 0.02).

^b^In comparison with the same period in 2019, the CA volume alterations in 2021 were not statistically significant (*p* = 0.49).

CA, catheter ablation; COVID-19, Coronavirus Disease 2019.

In contrast, a statistically insignificant increase in the CA volume was found in 2021 relative to 2019 (1.81%, 95% CI: −7.01 to 3.8%, *p* = 0.49). In addition, although the CA volume declined in January and February 2021 relative to 2019 as well, it increased rapidly in March 2021 relative to 2019 (Figures 1B,D). Meanwhile, unlike in 2020, a statistical insignificant inverse correlation (β = −945, 95% CI: −2784 to 893, *p* = 0.279) was observed between the monthly CA volume alterations in 2021 relative to 2019 and the monthly number of new COVID-19 cases in 2021 (Figure 2B), although there was still a total of 15,243 COVID-19 cases in 2021.

In total, there was a dramatic increase of 25.04% (95% CI: 7.55 to 42.53%, *p* = 0.006) in the CA volume in 2021 relative to 2020. Simultaneously, there was a decline in the annual number of COVID-19 cases in 2021 [15,243; 652 (520, 1,657)] relative to 2020 [87,071; 1,147 (452, 1,815)] (*p* = 0.02), mainly due to the disparity in January and February. There was no statistical discrepancy in COVID-19 cases over the remaining months between 2020 and 2021 after these 2 months were removed (*p* = 0.12).

### Regional CA volume alterations from 2019 to 2021

**Figure 3:**
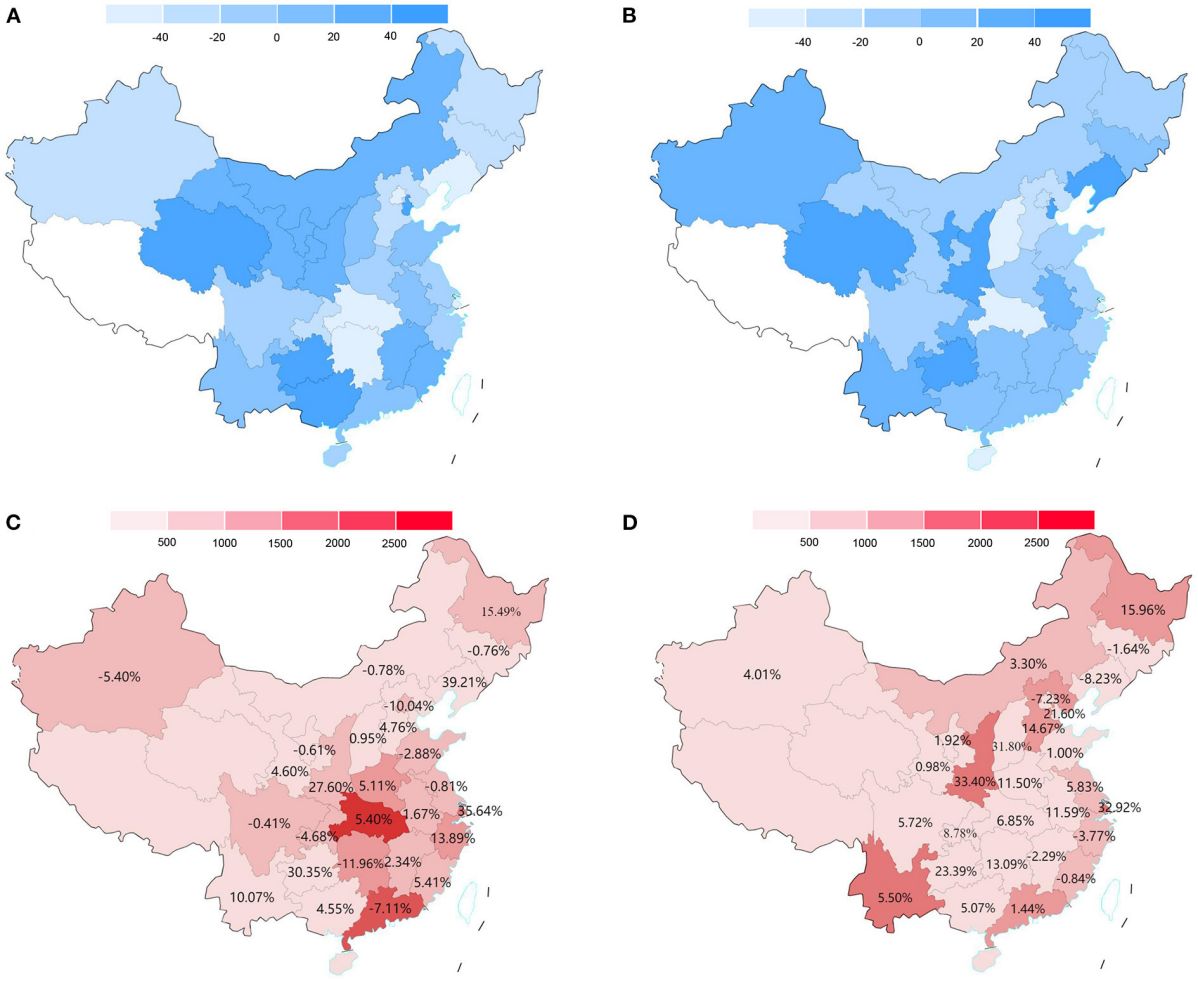
The regional CA volume alterations in 2020 or 2021 relative to 2019 and COVID-19 cases in 2020 and 2021. **(A)** China map for the percentage of CA volume alterations in each province in 2020 relative to 2019. **(B)** China map for the percentage of CA volume alterations in each province in 2021 relative to 2019. **(C)** China map for the annual number of COVID-19 cases in each province in 2020 and alterations in the proportion of CA in secondary hospitals in 2020 relative to 2019. **(D)** China map for the annual number of COVID-19 cases in each province in 2021 and alterations in the proportion of CA in secondary hospitals in 2021 relative to 2019. For sections A and B, the value >0 means that the CA volume is increased relative to 2019. Provinces (Tibet, Hong Kong, Macau, and Taiwan) lacking relevant data are filled with white. CA, catheter ablation; COVID-19, Coronavirus Disease 2019.

Compared with 2019, the CA volume in the majority (21/30) of the provinces decreased to varying degrees in 2020 (Figure 3A). The regional CA volume experienced a dramatic decline of 18.58% (95% CI: −31.33% to −6.11%, *p* = 0.001) in 2020 relative to 2019. In particular, the CA volume decreased by 51.86% in Hubei province with the highest COVID-19 cases in China in 2020. Moreover, there was a similar prominently inverse association with β of −1699 (95% CI: −2,391 to −1,007, *p* < 0.001) between annual CA volume alterations and the corresponding number of new COVID-19 cases in each province (Figure 2C).

However, the decline in CA volume was observed in a minority (11/30) of provinces (Figure 3B) and there was a non-significant increase (1.81%, 95% CI: −11.91% to 15.57%, *p* = 0.80) in CA volume in 2021 relative to 2019. Similarly, a statistically insignificant correlation (β = −309, 95% CI: −1057 to 438, *p* = 0.404) was found between the CA volume alterations in 2021 relative to 2019 and the corresponding number of new COVID-19 cases in 2021 in each province (Figure 2D).

In total, the regional CA volume increased significantly by 25.04% (95% CI: 15.23 to 32.24%, *p* < 0.001) in 2021 relative to 2020. The annual regional number of COVID-19 cases in 2020 and 2021 were shown in Figures 3C,D. A total of 87,071 [M (IQR), 551.5 (228.5, 988.5)] and 15,243 [M (IQR), 311.0 (37.50, 872.8)] COVID-19 cases were reported in 2020 and 2021, respectively. The difference in regional COVID-19 incidence between 2020 and 2021 mainly due to the discrepancy in Hubei provinces [2020 vs 2021, 78.27% (68,149/87,071) vs. 1.10% (168/15,243)]. No similar distribution differences in COVID-19 cases were found in 2021, and the province with the highest COVID-19 cases was the Shaanxi province (1712).

### The redistribution of CA in different levels of hospitals from 2019 to 2021

**Figure 4:**
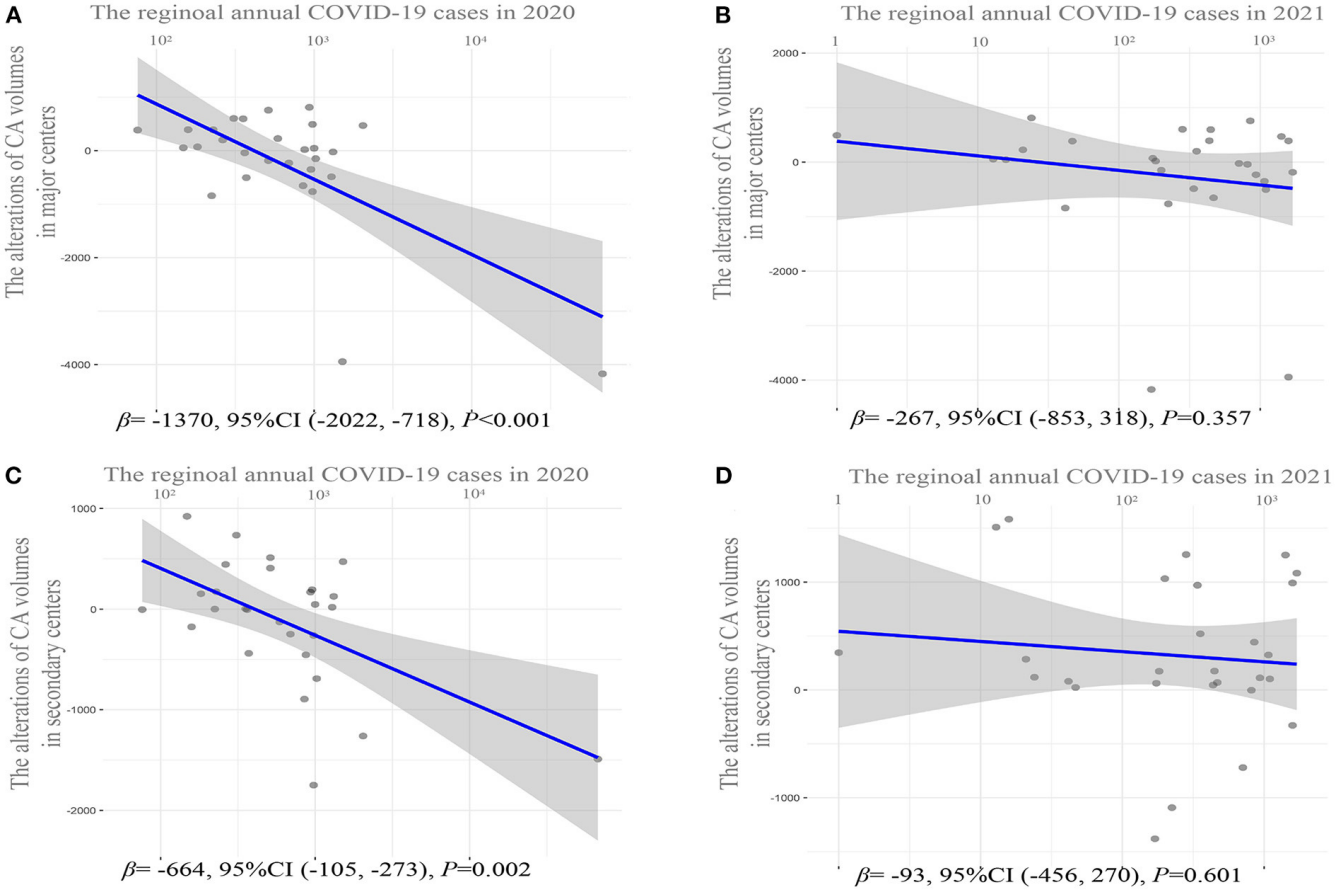
Correlation analysis of regional CA volume alterations in major centers or secondary centers in 2020 or 2021 relative to 2019 and the corresponding number of new COVID-19 cases. **(A)** Scattered plot for CA volume alterations in major centers in 2020 relative to 2019 and the corresponding number of new COVID-19 cases in each province in 2020. **(B)** Scattered plot for CA volume alterations in major centers in 2021 relative to 2019 and the corresponding number of new COVID-19 cases in each province in 2021. **(C)** Scattered plot for CA volume alterations in secondary centers in 2020 relative to 2019 and the corresponding number of new COVID-19 cases in each province in 2020. **(D)** Scattered plot for CA volume alterations in secondary centers in 2021 relative to 2019 and the corresponding number of new COVID-19 cases in each province in 2021. The major centers denote the top three hospitals according to the CA volume in each province. The secondary centers denote hospitals other than the top three hospitals in each province. CA, catheter ablation; COVID-19, Coronavirus Disease 2019.

Table 2 details the proportion of CA volume in the top 50 hospitals nationwide and the major centers in each province in 2019, 2020, and 2021. In 2020, both the proportion of CA volume in the top 10 hospitals (19.06vs. 22.01%) and the top 50 hospitals (47.20 vs. 51.95%) experienced a marked decline relative to 2019 (*p* < 0.001). Moreover, both the number and proportion of CA volume in the major centers declined dramatically in 2020 relative to 2019 (72,392 vs. 52,777; 53.26 vs. 59.28%, *p* < 0.001). Simultaneously, whereas the CA volume (46,302 vs. 49,719, *p* < 0.001) in the secondary centers declined as well, the proportion of the CA volume in the secondary centers (Figure 3C) increased significantly (46.73 vs. 40.71%, *p* < 0.001). In addition, there was a significant inverse correlation between regional CA volume alterations in major centers (β = −1370, 95% CI: −2022 to −718, *p* < 0.001) and secondary centers (β = −664, 95% CI: −105 to −273, *p* = 0.002) in 2020 relative to 2019 and the corresponding number of new COVID-19 cases in 2020, which means that the annual CA volume decreased by 1,370 and 664 in major centers and secondary centers, respectively, on average per 10-fold increase in the annual number of COVID-19 cases (Figures 4A,B).

**Table 2:** The CA volume alterations in the top 50 hospitals and major centers during the pandemic.

**–**	**2019**	**2020**	**2021**
**Integral analysis**	–	–	–
1st−10th	27,032 (22.01%)	19,062 (19.06%)^a^	21,559 (17.4%)^a, b^
11th−20th	12,332 (10.04%)	10,762 (10.76%)^a^	13,984 (11.18%)^a, b^
21st−30th	9,770 (7.95%)	6,412 (6.41%)^a^	8,091 (6.47%)^a, b^
31st−40th	8,264 (6.73%)	7,043 (7.04%)^a^	5,018 (4.01%)^a, b^
41st−50th	6,413 (5.22%)	3,930 (3.93%)^a^	5,235 (4.19%)^a, b^
1st−50th	63,811 (51.95%)	47,209 (47.20%)^a^	53,887 (43.09%)^a, b^
After 50th	58,631 (48.05%)	52,810 (52.80%)^a^	71,179 (56.91%)^a, b^
**Fractional analysis**	–	–	–
Major centers	72,392 (59.28%)	52,777 (53.26%)^a^	66,152 (52.73%)^a, c^
Secondary centers	49,719 (40.71%)	46,302 (46.73%)^a^	58,914 (47.27%)^a, c^

The top 50 hospitals denote the top 50 hospitals nationwide by CA volume.

The major centers denote the top three hospitals according to the CA volume in each province.

The secondary centers denote hospitals other than the top three hospitals in each province.

^a^denotes the *p* < 0.05 relative to 2019.

^b^denotes the *p* < 0.05 relative to 2020.

^c^denotes the *p* = 0.08 relative to 2020.

CA, catheter ablation.

There was a statistically insignificant increase in CA volume in 2021 relative to 2019 (125,006 vs. 122,839, *p* = 0.80). However, the proportion of CA volume in the top 10 hospitals (17.24 vs. 19.06%) and top 50 (43.09 vs. 47.20%) hospitals nationwide further declined in 2021 relative to 2020 (*p* < 0.001). Although the further downward trend in the proportion of CA volume in major centers in 2021 relative to 2020 was statistically insignificant (Table 2). It is noted that both the number and proportion of CA volume in secondary centers (Figure 3D) increased significantly in 2021 relative to 2019 (58,914 vs. 49,719; 47.27 vs. 40.71%, *p* < 0.001). Meanwhile, there was no significant inverse correlation between CA volume alterations in either major or secondary centers in 2021 relative to 2019 and the corresponding number of new COVID-19 cases in 2021 (Figures 4C,D).

## Discussion

To the best of our knowledge, this is the first study to depict a nationwide insight concerning the successive impact of the COVID-19 pandemic on CA with data from the NCCQI platform. The CA volume declined substantially in the early phase of the COVID-19 pandemic, and then gradually returned to pre-COVID-19 levels. Meanwhile, a significantly negative correlation was found between the CA volume alterations and the corresponding number of new COVID-19 cases in 2020, but not in 2021. In addition, the growth of CA volume in secondary hospitals played an important role in the overall resumption process. Our findings indicate that systematic guidance for secondary hospitals with CA experience may conduce to mitigate the negative impact of the COVID-19 pandemic.

Previous studies in several countries have reported the negative impact of the COVID-19 pandemic on CA primarily in the early days of the outbreak. The CA volume declined by 21% in Germany between March 1, 2020, and April 30, 2020, relative to the same period in 2019 (10). A British study with data from a nationwide administrative database found a significant decline in AF ablation (−83%) and other ablations (−64%) from 23 March 2020 to 26 July 2020 relative to the previous year's control (7). Consistent with the above publications, we found that the CA volume experienced a maximal reduction of 88.78% in February 2020 corresponding with the peak of the monthly number of new COVID-19 cases, and a significant decline of 54.32% during the 3-months lockdown.

Three reasons may account for the remarkable decline in CA volume in the early stages of the outbreak. First, to minimize the spread of the COVID-19 pandemic, many countries enforced rigorous public strategies based on staying home, limiting social activities, and even blockade of pandemic areas, which inevitably exerted a negative impact on the routine management of non-communicable diseases (3, 5, 20, 21). Second, the limited medical resources were reorganized to restrain the spread of the pandemic and ensure the response to emergency cases. Consequently, the majority of non-urgent CA have been postponed (7, 22). Third, the reduction in medical visits for fear of nosocomial infection further reduced the detection rate of patients with CA indications (23–25).

Our findings showed that the CA volume in China declined significantly between January and March 2020 when public health measures were most stringent and new confirmed COVID-19 cases were at the peak. In addition, our study provides additional information regarding the subsequent changes in CA volume. As the number of newly confirmed COVID-19 cases declined and restrictions were gradually downgraded, the monthly CA volume increased firstly in June 2020 relative to the same period in 2019. Since then, the CA volume in 2020 remained unchanged relative to the same period in 2019. Although the growth was not significant enough relative to 2019, there was a dramatic increase in CA volume in 2021 relative to 2020.

Multiple factors may promote the gradual recovery of CA in China. Firstly, the dynamic, scientific, and rigorous anti-COVID-19 strategies which were implemented by the government and strictly followed by residents played an indispensable role in controlling the spread of the pandemic (24, 26). Secondly, the COVID-19 vaccine, which may both reduce the risk of infection and fear of the pandemic, has been extensively available since January 2021 in China (27, 28). Thirdly, the growth of CA volume in secondary hospitals may be another important motivator.

In 2020, CA in major centers was more affected by the corresponding number of new COVID-19 cases than that in the secondary centers. In addition, despite the CA volume increasing by 2227 in 2021 relative to 2019, the CA volume still decreased by 9,924 in the top 50 hospitals nationwide and by 62,40 in the major centers in each province. Besides the objective restrictions on movement resulted in patients having to go to local hospitals. The “3R Telemedicine Project” jointly launched by the CSA and CSPE aiming to improve the capacity of secondary centers may have actively promoted the redistribution of CA. This would have occurred especially in 2021, when either the decline in CA volume in major centers or the growth of CA volume in secondary centers was not significantly associated with the corresponding number of new COVID-19 cases. Our findings revealed the enormous potential of secondary hospitals with CA experience under systematic guidance in coping with the negative impact of the COVID-19 pandemic. Considering that the COVID-19 pandemic is still ongoing worldwide, our findings may have vital implications for other countries in promoting the recovery of CA.

Despite the lack of a comparative study about the subsequent impact of the COVID-19 pandemic on CA, a repeated wave descent in 2020 in England (29) and continuous decline between March 2020 and March 2021 relative to pre-COVID-19 in Israel (30) in myocardial infarction hospitalizations were reported. Given that CA had been more significantly affected than medical care for myocardial infarction by the pandemic, the gradual recovery of CA observed in China may not be a global trend (7). Delays in the emergency intervention of acute conditions such as myocardial infarction resulted in increased mortality (3–5). Simultaneously, the detriment of postponing these non-acute procedures would also be foreseeable in the near future. For example, pulmonary vein isolation can achieve more than 90% success in patients with paroxysmal AF (31). However, for patients with longstanding persistent AF, an additional left atrial substrate modification is often essential and with a reduction of 55% in sinus rhythm maintenance in the 2-year follow-up (32). It has been reported that around 6–50% of patients with paroxysmal AF progress to persistent AF annually (33) and around 35–40% of patients with persistent AF progress to permanent AF within 1 year (34). Hence, the postponement of elective procedures could be reasonable to mitigate the spread of the pandemic during the outbreak. Still, a timely reboot with rigorous precautions should be considered.

Our findings should be interpreted within the context of several potential limitations. Firstly, the nature of the observational study design restrains causal inference; however, a similar downward trend is consistent with other published studies in different countries (7, 10). Secondly, our study is based on an administrative rather than a clinical database. Despite having access to nationwide data, detailed information regarding patient demographic characteristics and procedures for urgent or elective procedures was unavailable. Thus, failing to further explore the independent impact of the COVID-19 pandemic on CA. But in fact, there may be a few factors that could have the same level of impact as the pandemic. Thirdly, the recovery of CA in China may be partially attributed to the consistent and rigorous anti-COVID-19 strategy; thus, the generalizability of our findings remains unclear. Further studies are needed to determine whether the recovery of CA is global to deal with the future adverse consequences due to today's decline.

## Conclusions

The CA volume declined significantly in the early phase of the COVID-19 pandemic, and then gradually returned to pre-COVID-19 levels. Notably, the growth of CA volume in secondary hospitals plays an important role in the overall resumption process, which implies that systematic guidance for secondary hospitals with CA experience may conduce to mitigate the negative impact of the COVID-19 pandemic. Our findings may have vital implications that can be applied in responding to and preparing for current and future pandemics.

## Data availability statement

The raw data supporting the conclusions of this article will be made available by the authors, without undue reservation.

## Author contributions

SZhan had the idea for the study and contributed to the study design. JJ and SZhao collected the data, conducted a literature review, and drafted the manuscript. XN and CC provided guidance and support for statistical analysis. NL and PL interpreted data and coded the figures. XN is responsible for managing the NCQQI platform. All authors contributed to the study design, literature review, data analysis, manuscript writing, and revision. All authors have read and approved the final manuscript.

## Funding

This work was supported by the Natural Science Foundation of China (81470466), the National Science and Technology Pillar Program during the 12th Five-Year Plan Period (2011BAI11B02), and Beijing Municipal Science and Technology Commission (Z191100006619120). The funders had no role in the study design, data collection, analysis, or decision to publish.

## Conflict of interest

The authors declare that the research was conducted in the absence of any commercial or financial relationships that could be construed as a potential conflict of interest.

## Publisher's note

All claims expressed in this article are solely those of the authors and do not necessarily represent those of their affiliated organizations, or those of the publisher, the editors and the reviewers. Any product that may be evaluated in this article, or claim that may be made by its manufacturer, is not guaranteed or endorsed by the publisher.

## Abbreviations

AF: atrial fibrillation
AFL: atrial flutter
AT: atrial tachycardia
CA: catheter ablation
COVID-19: Coronavirus Disease 2019
CI: confidence interval
CSA: Chinese Society of Arrhythmia
CSPE: Chinese society of pacing and electrophysiology
NCCQI: National Center for Cardiovascular Quality Improvement
PSVT: paroxysmal supraventricular tachycardia
PVC: premature ventricular contraction
VT: ventricular tachycardia
3R: Tri-Remote.
